# Role of dose selection in successful interleukin-2 immunotherapy: solving the Goldilock’s Complex

**DOI:** 10.1186/2051-1426-1-S1-P266

**Published:** 2013-11-07

**Authors:** Andrew Zloza, Joseph R Broucek, Erica J Huelsmann, Tasha Hughes, Howard L Kaufman

**Affiliations:** 1 Immunology/Microbiology, Rush University Medical Center, Chicago, IL, USA; 2 General Surgery, Rush University Medical Center, Chicago, IL, USA

## Background

The results of multiple IL-2 clinical trials (using a range of doses) have shown similar trends, in which IL-2 immunotherapy leads to an objective response in 15-20% of patients and complete response in 5-10%. Importantly, the majority (> 80%) of patients with a complete response maintain long-term responses. However, why only a small proportion of patients attain a complete response at any dose is unknown. We hypothesized that the dose of IL-2 utilized, differentially targets CD8+ effector versus CD4+ regulatory T cells based on patient-specific characteristics, and that an optimal IL-2 dose may be needed to achieve therapeutic benefit.

## Methods

C57BL/6 mice (10 per group) were adoptively transferred (via retroorbital injection) with pmel CD8+ T cells (for tracking responses; 100,000 cells) and challenged with B16-F10 melanoma (intradermally with 120,000 cells) on day 0. Four groups were treated on days 5-9 via intraperitoneal injection with IL-2 (100,000, 10,000, or 1,000 units) or PBS only (placebo group). Tumor area was measured every other day for 30 days. Primary outcomes included tumor growth and T cell characterization.

## Results

Tumor growth at day 14 was significantly reduced with 10,000 and 100,000 units of IL-2 (31 and 38 mm2, respectively) compared to the placebo, (61 mm2) whereas surprisingly, it was significantly increased with 1,000 units (96 mm2) compared to placebo (P<0.02 for all doses versus placebo). While the number of CD8+ T cells, CD4+ T cells, and NK cells in the tumor were similar in all groups tested, the ratio of pmel CD8+ T cells compared to CD4+ Tregs was increased with the 10,000 and 100,000 unit doses, and decreased with the 1,000 units dose when compared to the placebo. Unexpectedly, the 100,000 unit dose resulted in fewer pmel CD8+ T cells compared to 10,000 unit dose.

## Conclusions

An optimal dose of IL-2 (here, 10,000 units) significantly decreased tumor growth and increased overall survival when compared to non-optimal lower or higher doses of IL-2 in an animal melanoma model. We are now exploring how IL-2 dose range varies among patients and may impact clinical outcomes. Ultimately, these findings are expected to improve therapeutic responses and identify measurable biomarkers to optimize IL-2 dosing that can be personalized to each patient undergoing IL-2 immunotherapy.

